# Single-cell RNA sequencing and proteomics uncover SIRPα-CD47 immune checkpoint and glycolysis-driven immune evasion in cardiac myxoma

**DOI:** 10.3389/fimmu.2025.1652645

**Published:** 2025-09-01

**Authors:** Xinyuan Zhu, Qingle Gao, Xintong Dai, Baofa Sun, Yanping Li, Hongyan Zhai, Changliang Shan

**Affiliations:** ^1^ Department of Cardiovascular Surgery, The Fifth Central Hospital of Tianjin, Tianjin, China; ^2^ State Key Laboratory of Medicinal Chemical Biology, College of Pharmacy and Tianjin Key Laboratory of Molecular Drug Research, Nankai University, Tianjin, China; ^3^ Tianjin Pharmaceutical Research Institute Co., LTD, Tianjin, China; ^4^ State Key Laboratory of Medicinal Chemical Biology, College of Life Science, Nankai University, Tianjin, China; ^5^ Department of Pathology and Institute of Precision Medicine, Jining Medical University, Jining, China; ^6^ Department of Ultrasound, Tianjin Medical University General Hospital, Tianjin, China

**Keywords:** single-cell sequencing, immune microenvironment, glycolysis, immune evasion, M2 polarization, IL-10

## Abstract

Cardiac myxoma (CM), a rare primary cardiac tumor, poses significant life-threatening risks. Current CM research has remained largely limited to clinical case observations and pathological analyses, thus restricting its clinical therapeutic impact. Fundamental research should be urgently strengthened to better support future CM treatment strategies. In this work, single-cell sequencing is used to elucidated the intricate cellular composition of the CM microenvironments. The mechanisms of heart myxoma cell growth are investigated via proteomics and organoid models, while our western blot analysis reveals cardiac myxoma’s immune evasion strategies. This study successfully characterizes diverse cell types within the CM microenvironment. Notably, ap-CAF cells are found to effectively recruit immune cells via chemokine secretion, fostering immune microenvironment formation. The work’s pseudotime trajectory analysis also demonstrates that CM tumor cells derive from mesenchymal stem cells. Additionally, this work demonstrated that the glycolysis pathway is significantly activated and fuels CM cell growth. Tumor cells exploit the SIRPα-CD47 immune checkpoint to evade the immune system by inhibiting antigen-presenting cell phagocytosis. Tumor-associated macrophages (TAMs) concurrently assume M2 polarization and suppress autoimmune activity through *IL-10*. This research comprehensively examines CM’s microenvironmental cellular architecture, metabolic features, and immune escape mechanisms. These work’s findings not only deepen the current understanding of CM’s biological nature but also offer vital theoretical foundations for developing safer, more effective CM therapies.

## Introduction

Primary cardiac tumors (CM) are exceptionally rare: Three-quarters of all CM are histologically classified as benign, predominantly myxomas. The annual incidence of CM is measured at approximately two cases per million individuals (1, 2). Although considered rare and benign, CM can cause significant morbidity through complications such as tumor stroma detachment, potentially causing vascular occlusion and cerebrovascular events (3). While surgical resection is currently the gold standard for CM management (4), this approach poses challenges for elderly high-risk patients, and its postoperative recurrence factors remain poorly understood. Advancing CM intervention strategies necessitates a comprehensive investigation into CM’s pathogenic mechanisms and developmental pathways.

Tumor research over recent years has increasingly recognized single-cell transcriptomic sequencing (scRNA-seq) applications in tumor research (5, 6). Notably, scRNA-seq has substantially propelled cardiovascular disease investigations. Felipe Prosper’s work detailed cardiac fibroblast heterogeneity and its dynamic behavior after myocardial infarction, reshaping our understanding of their role in injury response and myocardial remodeling processes (7). Ali J. Marian revealed through scRNA-seq that extracardiac fibroblasts and epithelial cells produce paracrine mediators that facilitate the epithelial–mesenchymal transition. In arrhythmogenic cardiomyopathy (ACM) models, these factors also promote myocardial fibrosis, apoptosis, and the development of arrhythmias and heart failure (8). Few studies have employed multiomics approaches to examine cardiac myxoma. The synergy between scRNA-seq and proteomics presents a robust methodology for exploring CM biology. scRNA-seq permits the characterization of cellular diversity within CMs and functional dynamics in their microenvironment (9–11). Thorough analysis of the tumor microenvironment and its constituents can deepen insights into CM pathogenesis and behavior (12). Multiomics investigations of cardiac myxoma currently remain scarce. Combining multiomics strategies enables the holistic examination of CM microenvironment features and disease mechanisms.

scRNA-seq and proteomic profiling were conducted in this work to uncover CM microenvironment properties and immune evasion tactics. We identified diverse cell populations within CM microenvironments. Notably, apCAFs were found to critically orchestrate immune cell recruitment via chemokine secretion (MIF, ANXA1); moreover, we discovered that despite abundant immune infiltration, tumor cells employ sophisticated evasion strategies. These tumor cells engage the SIRPα-CD47 axis to inhibit antigen-presenting cell phagocytosis, while glycolytic metabolites polarize TAMs toward M2 phenotypes, which secrete IL-10 to dampen immune responses. These coordinated mechanisms allow CM persistence despite immune surveillance.

## Materials and methods

The data, all protocols, and study materials will be available to other researchers for purposes of reproducing the results or replicating the procedures that are presented in this article.

### Human sample collection

Human CM tissues and adjacent non-tumor CM tissues were collected from Tianjin Medical University General Hospital (Tianjin, China) after surgical resection, for scRNA-seq, proteomics, organoid model, and histologic analysis. The clinical CM specimens all have the written consent approving the use of the samples for research purposes from patients. The relevant characteristics were shown in **Supplementary Table 1**. The study protocol was approved by Tianjin Medical University General Hospital Ethics Committee.

### CM tissue single-cell processing

CM tissues from three patients (P1, P2, P3) and adjacent non-tumorous tissues from two patients (P1, P3) were subjected to scRNA-seq. After harvesting fresh tissues by surgery, using ice-cold RPMI1640 and Multi-tissue dissociation kit 2 wash and dissociate the tissues as instructions. After removing erythrocytes (using Miltenyi 130-094-183), cell count and viability were assessed using a fluorescence cell analyzer (Countstar^®^ Rigel S2) with AO/PI reagent. According to the results, choose whether to perform debris and dead cell removal using Miltenyi 130-109-398/130-090-101. Finally, the fresh cells were washed twice in RPMI1640, resuspended at a concentration of 1×10^6^ cells per ml in 1×PBS, and supplemented with 0.04% bovine serum albumin.

### 10× genomics chromium library preparation and sequencing

Single-cell RNA-Seq libraries were prepared with SeekOne^®^ Digital Droplet Single Cell 3’ library preparation kit. Single-cell separation and capture were achieved by water-in-oil, and nucleic acid modified Barcoded Beads were used for molecular labeling of RNA from different cells. Then high-throughput single-cell transcriptome libraries compatible with Illumina NovaSeq 6000 sequencers were constructed. Finally, the SeekOne^®^DD single-cell 3 ‘transcriptome Kit was sequenced using Illumina NovaSeq 6000 high-throughput sequencing platform with ≥50,000 reads per cell to ensure the accuracy of the single-cell sequencing data analysis.

### Single-cell RNA quality control and sequencing analysis

SeekOne^®^ Tools were used to perform quality control on the raw sequencing data. Subsequently, Seurat (v4.1.1) was utilized for downstream single-cell sequencing analysis. Cells with over 200 expressed genes and a mitochondrial unique molecular identifier (UMI) rate below 10% passed the cell quality filtering, and mitochondrial genes were removed from the expression table. A total of 55793 met the above quality control requirements.

### Differentially expressed genes and function enrichment analysis

The FindAllMarkers function within the Seurat R package is employed to investigate differentially expressed genes (DEGs) across various cell types, clusters, and groups. Log-transformed fold change threshold greater than 0.5, ‘min.pct’ greater than 0.25, and p-value less than 0.05 were considered indicative of significantly different genes.

ClusterProfiler (v4.2.0) R package was used to perform gene enrichment analysis using the msigdbr database37. The items of pathway with enrichment score greater than 1 and a p-value less than 0.05 were deemed significant. In addition, the self-constructed gene set (**Supplementary Table 2**) according to previous studies was scored through the AddModuleScore function in Seurat R package or AUCell (v2.4.0).

Visualization was realized through ggplot2 (v3.3.6), jjAnno (v0.0.3) and Seurat with its own visualization function.

### Cell communication analysis

We performed cell-cell communication analysis with the cellchat software (v1.6.1). According to the Secreted Signaling and Cell-Cell Chat databases, cellchat was used to evaluate the interactions among cells, and we conducted an assessment of the signal intensity transmitted and received by every celltype. The receptor-ligand pairs (SIRPA-CD47) was added to database to fulfill the requirements of analysis. Meanwhile, Circle plots and heatmaps visualized the relationship between the ligands of interest, while violin plots visualized the expression levels of the ligand and receptor.

### Single-cell metabolism analysis

Based on the KEGG metabolic database, we used scMetabolism (v0.2.1) to assess the metabolic pathways across various samples. The resulting metabolic scores was visualized with heatmap.

### Cell trajectory analysis

The monocle (v2.2.6) package was used to analyze tumor cells trajectories. We used differentially expressed genes identified by Seurat to sort cells in pseudo-time order. The visualization functions ‘plot cell trajectory’ or ‘plot genes in pseudotime’ were used to plot the minimum spanning tree on cells.

### Evaluation of cell type purity

We utilized ROGUE (v1.0.0) to assess the purity of cell populations within each cluster. By default, a ROGUE score exceeding 0.9 indicates a highly consistent celltype.

### HE staining

HE Staining is one of the principal stains in pathology histology. We used the Hematoxylin-Eosin Stain Kit (Solarbio G1120) to observe the morphology of CM tissue samples and explore the composition of extracellular matrix and tumor microenvironment cells.

### Cell culture

THP-1 is a human monocytic cell line derived from an acute monocytic leukemia patient. The THP1 cells were cultured in RPMI1640 supplemented with 10% fetal bovine serum (FBS) and 0.05mM β-Mercaptoethanol. HEK293T cells were cultured in DMEM with 10% FBS. All cells were cultured at 37°C in an incubator supplied with 5% CO_2_.

### Inducing M0 macrophages *in vitro*

After thawing and culturing THP-1 cells in RPMI-1640 complete medium (supplemented with 0.05 mM β-mercaptoethanol) until they reached logarithmic growth phase, the cells were centrifuged at 800 rpm for 3 minutes. The pellet was resuspended in a measured volume of RPMI1640 complete medium containing 0.1% PMA, adjusted to a density of 2.5×1^5^ cells/mL, and seeded into 6-well plates at 500,000 cells per well. Following 24 hours of incubation, the old medium was discarded, and adherent cells were washed twice with 1 mL PBS per well before fresh RPMI1640 complete medium (with 0.1% PMA) was added for another 24-hour culture period. Microscopic examination after this second incubation confirmed that the majority of THP-1 cells had adhered and differentiated into M0-type macrophages. Finally, the medium was discarded, non-adherent cells were removed by PBS washing, and fresh RPMI1640 medium was introduced for subsequent experimental treatments.

### Conditioned medium treatment of M0 macrophages

Organoids treated with drugs were subjected to a process where the drug-containing medium was removed and replaced with fresh medium for further culturing over 48 hours. The organoid culture medium was then collected, centrifuged, aspirated, and transferred to a new tube with detailed sample information and time noted. Subsequently, a conditioned medium was prepared by mixing the collected media with fresh media at a 3:7 ratio. Following this, the old medium from culturing M0 macrophages was replaced in a 6-well cell culture plate with the conditioned medium, and the plate was placed in a cell culture incubator for 48 hours after proper documentation. Post-treatment, cell pellets were collected for lysis and protein samples were prepared for Western blot experiments to detect the expression of the target protein.

### Proteomics

CM tissues and adjacent non-tumorous tissues from patients P6, P7, and P8 were used for proteomic analysis. The tissue sample was frozen with liquid nitrogen, followed by lysis solution containing inhibitors. The tissue was then ground with a cold grinder at -35°C, and the supernatant was centrifuged to obtain a total protein solution. After measuring the concentration, the solution was aliquoted and stored. Each sample was taken at 50 μg, the concentration was adjusted, DTT was added and incubated, followed by the addition of iodoacetamide and cooling. Acetone was added to precipitate the protein, the supernatant was discarded after centrifugation, the precipitate was collected and acetone was evaporated. The precipitate was dissolved with TEAB, trypsin was added for overnight digestion, followed by freeze-drying and storage. For peptide labeling and detection, TEAB buffer was added to the freeze-dried sample, mixed well, TMT reagent was added for labeling reaction, the reaction was terminated, and freeze-dried for storage. Finally, peptides were identified using chromatography and mass spectrometry.

### Differential analysis

Differential expression proteins were identified based on Log2 (Fold Change) ≥ 1 or Log2 (Fold Change) ≤ -1 and p-value < 0.05 from t-test.

### Western blot assay

The cells were harvested and lysed using RIPA lysis buffer at 4°C. Following centrifugation, the protein concentration was determined with an Ultra Trace Ultraviolet Spectrophotometer. Equal amounts of cell lysate from each sample were then loaded onto SDS-PAGE. The proteins were subsequently transferred onto PVDF membranes (Millipore, USA), blocked with 5% skim milk, and incubated with antibodies targeting PKM2, PFKP, ENO1, TPI1, IL-10, β-actin etc. HRP-conjugated Goat Anti-mouse or Anti-rabbit IgG served as the secondary antibody. The signal was visualized using the Immobilon Western HRP Kit (Millipore, USA).

### Patient-derived organoids construction

​Patient-derived primary tissues from P17 were utilized for organoid generation. Collect primary tissue blocks and place them in a protective solution. Prepare the 24-well plate and 96-well plate required for the experiment, and preheat the culture medium and BME gel. Trim the tumor tissue under sterile conditions, remove non-epithelial components, cut into small pieces, and wash by centrifugation with a cleaning solution. Prepare a working solution of digestive enzymes and proceed with tissue digestion. Stop the digestion process at the appropriate time and centrifuge to collect the precipitate. If red blood cells are present, lyse them and centrifuge again. Resuspend cells in organoid culture medium, filter them through a cell strainer, and centrifuge once more. Resuspend the cells in a mixture of BME gel and culture medium at a ratio of 3:1, then quickly dispense it onto the preheated well plate, allowing it to solidify before adding more culture medium. Monitor and change the culture medium every 2–3 days while observing the growth of the organoids.

### CellTiter-Glo^®^ 3D Cell Viability Assay

The CellTiter-Glo^®^ 3D Cell Viability Assay is utilized for assessing the viability of cells cultured in a three-dimensional (3D) environment through the measurement of adenosine triphosphate (ATP) levels, which serve as an indicator of cellular metabolic activity. The unique composition of the CellTiter-Glo^®^ 3D Cell Viability Assay reagent is tailored to efficiently lyse cells, specifically optimized for evaluating the viability of microtissues generated in 3D cell culture settings. Notably, this assay obviates the requirement for cell washing, medium removal, or intricate pipetting procedures, as a singular application of the CellTiter-Glo^®^ 3D reagent enables uniform testing, compatible with serum, across various multi-well plate configurations, making it suitable for high-throughput screening applications. The CellTiter-Glo^®^ 3D assay kit has been effectively employed in discerning the viability of 3D microtissues cultured using diverse methodologies, such as cell hanging-drop plates, ultra-low attachment cell culture plates, Matrigel™-coated cell culture plates, agarose-coated cell plates, methylcellulose-covered culture medium, and 3D microtissues cultivated within Alvetex^®^ three-dimensional cell culture systems.

### Plasmid construction

The procedure of plasmid assembly involves the selection and design of plasmids, enzymatic digestion and ligation, bacterial transformation, screening and cultivation, as well as plasmid extraction and verification. Initially, the target fragment is isolated from 293T cells using the REL sequence (Forward: CGGAATTCAATGGCCTCCGGTGCGTATAAC; Reverse: CGGGATCCTTATACTTGAAAAAATTCATATGGAAAGGAGTCACTC), and the p3×FLAG-CMV plasmid is chosen with the design of the insert gene sequence. Subsequently, the insert fragment is connected to the plasmid backbone through enzymatic digestion and ligation. Following this, the ligated plasmid is introduced into bacteria for the identification of strains containing the recombinant plasmid. Lastly, plasmid DNA is isolated from the selected strains and confirmed to ensure the accuracy of plasmid construction.

### Analysis of c-REL Chip-seq data

After downloading the Chip-seq data of c-REL from the GEO database GSE55105, we loaded the reference genome of the corresponding species in IGV and import the properly formatted Chip-seq data. Following this, the IL-10 promoter region should be precisely located and the binding site of c-REL thoroughly analyzed to determine its binding status in this region. This step is essential for gaining a more comprehensive understanding of the specific role of c-REL in regulating IL-10 expression.

### Statistical analyses

In the statistical analysis, we used t-tests to compare significant differences in extracellular matrix and chemokine gene set scores among different fibroblast cell populations. Additionally, we conducted t-test analyses on the expression levels of the transcription factor REL in various cell groups to assess the significance of its expression levels. Statistical significances were indicated by *P<0.05, **P<0.01, ***P<0.001.

### Code availability

Custom-written scripts used in this study are available from the corresponding author on reasonable request.

## Results

### Single-cell analysis reveals the transcriptomic landscape in CM

**Figure 1:**
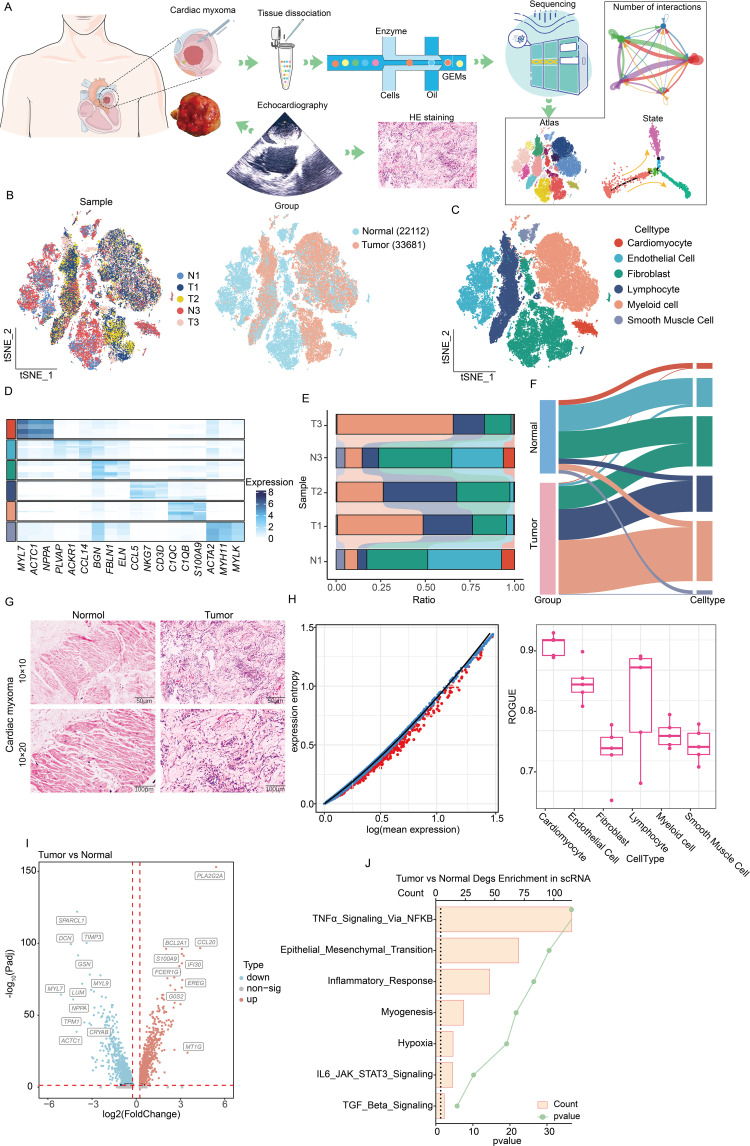
Single-cell sequencing analysis of the composition of the microenvironment of CM. **(A)** Experimental design and workflow of scRNA-seq of CM. **(B)** Integration of single-cell sequencing samples for t-SNE dimensionality reduction plot. **(C)** t-SNE dimensionality reduction plot shows the infiltrating cell types in the microenvironment of CM. **(D)** Heatmap showing expression levels of specific markers in each cell type. **(E)** Histogram represents the proportional distribution of different cell types in different tissues and samples. **(F)** Sankey diagram showing the distribution of different cell populations. **(G)** HE staining reveals the pathological characteristics of tumor tissue and adjacent tissue. **(H)** Using the ROGUE algorithm to calculate the purity of infiltrating cells in the microenvironment of cardiac myxoma. **(I)** The volcano plot shows the differential gene expression between tumors and adjacent tissues. **(J)** The bar chart displays the enrichment of differential genes between two groups. Identical colors denote the same cell type in **(C-F)**.

To elucidate CM pathogenesis and tumor microenvironment features, we enrolled echocardiography-confirmed left atrial CM patients for single-cell sequencing (**Figure 1A**). Following stringent quality control, 55,793 cells (13,829 paratumors; 21,109 tumors) were processed using Seurat (13) (**Figure 1B**). Subsequent analyses identified 24 clusters, with Seurat revealing six cell types: cardiomyocytes (*MYL7*, *ACTC1*, *NPPA*), endothelial cells (*PLVAP*, *ACKR1*, *CCL14*), fibroblasts (*BGN*, *FBLN1*, *EUN*), lymphocytes (*CCL5*, *NKG7*, *CD3D*), myeloid cells (*C1QC*, *C1QB*, *S100A9*), and smooth muscle cells (*ACTA2*, *MYH11*, *MYLK*) (**Figures 1C, D**). The cellular distribution analysis demonstrated a distinct composition between CM and paratumor tissues: Immune cells (lymphocytes, myeloid cells) indicated greater tumor infiltration, while endothelial and smooth muscle cells predominated in adjacent tissues. Moreover, fibroblasts were distributed in both regions (**Figures 1E, F**). HE staining validated these microenvironmental differences (**Figure 1G**). Our ROGUE score (14) assessment revealed high purity in cardiomyocytes and endothelial cells (scores of >0.8), with other types indicating lower purity (**Figure 1H**). In addition, the differential gene analysis conducted in this study identified upregulated pathways in CM (TNFα-NFκB signaling, EMT, and TGFβ signaling), suggesting their potential roles in CM pathogenesis (**Figures 1I, J**). Collectively, these findings demonstrate distinct cellular and molecular profiles between CM and paratumor microenvironments.

### scRNA-seq reveals the origin and functional characteristics of CM

**Figure 2:**
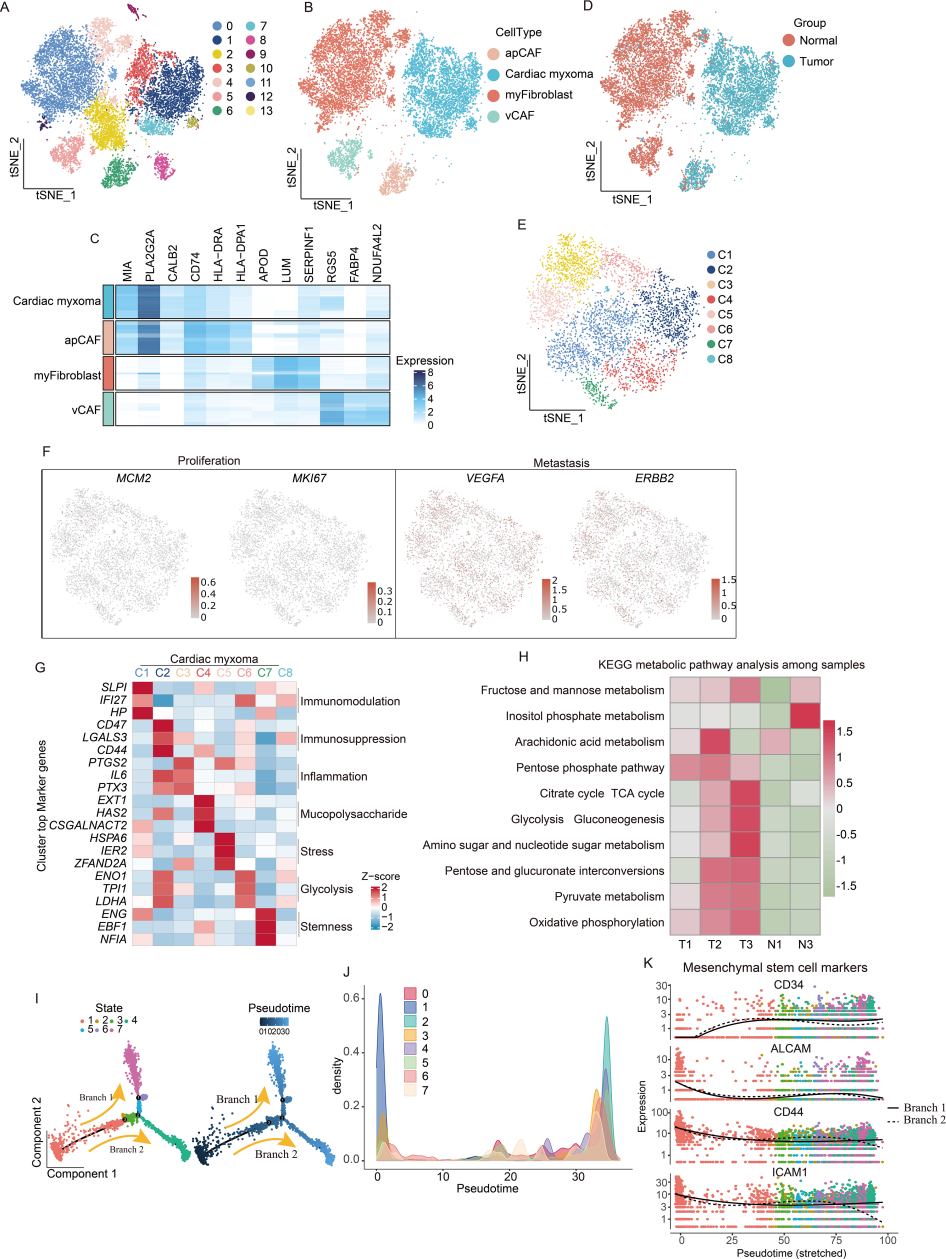
scRNA-seq reveals functional heterogeneity and origin of tumor cells. **(A)** Demonstration of the extraction, clustering, and dimensionality reduction effects of fibroblast cells. **(B)** t-SNE dimensionality reduction plot showing cell cluster annotation results. **(C)** Heatmaps are utilized to visually represent the expression levels of specific marker genes associated with four distinct subtypes of fibroblast cells. **(D)** t-SNE dimensionality reduction plot showing the distribution of fibroblast subpopulations. **(E)**, t-SNE dimensionality reduction plot shows that tumor cells have been extracted and divided into 8 clusters. **(F)** Featureplot displaying the gene score of proliferation and metastatic markers. **(G)** Analysis of the characteristic genes of each population of tumor cells reveals their functional heterogeneity. **(H)** KEGG metabolic pathway analysis reveals metabolic characteristics of CM. **(I)** pseudotime trajectory tree of all the tumor cells with different colors indicating different clusters, with darker colors indicating earlier appearance. **(J)** Temporal analysis reveals clustering situations. **(K)** Expression of mesenchymal stem cells marker genes in pseudotime branches.

Our initial observations revealed that fibroblasts displayed broad tissue distribution and comparatively low purity (**Figure 1H**). To characterize fibroblast subtypes, we performed extraction, dimensionality reduction, and clustering. The conducted differential gene analysis distinguished three tumor-associated fibroblast populations: apCAF, vascular CAFs (vCAF), and myofibroblasts (**Figures 2A–C**). Cellular distributions differed significantly between tumor and adjacent tissues—apCAFs and tumor cells localized predominantly in tumor regions, while myofibroblasts and vCAFs were enriched in paratumor areas (**Figure 2D**). Further tumor cell subclustering yielded eight distinct groups (**Figure 2E**). All clusters showed restricted proliferative and metastatic potential based on *MCM2*, *MKI67*, *VEGFA*, and *ERBB2* expression—consistent with the benign nature of CM (**Figure 2F**). Cluster-specific gene analysis revealed unique functional specializations during CM progression (**Figure 2G**), although all maintained active glucose and amino acid metabolism (**Figure 2H**). Given the fibroblast-like properties of tumor cells and their shared mesenchymal stem cell (MSC) origin with fibroblasts, we performed pseudotime analysis using MSC markers (15). This temporal clustering allowed us to organize tumor cells into seven developmental stages (**Figures 2I, J**). Our tracking of MSC marker expression revealed progressive CD34 upregulation alongside declining *ALCAM*, *CD44*, and *ICAM1* levels (16) (**Figure 2K**), indicating complex transcriptional reprogramming during MSC-to-tumor cell transitions.

### Proteomics combined with organoid model reveals vigorous glycolysis in CM

**Figure 3:**
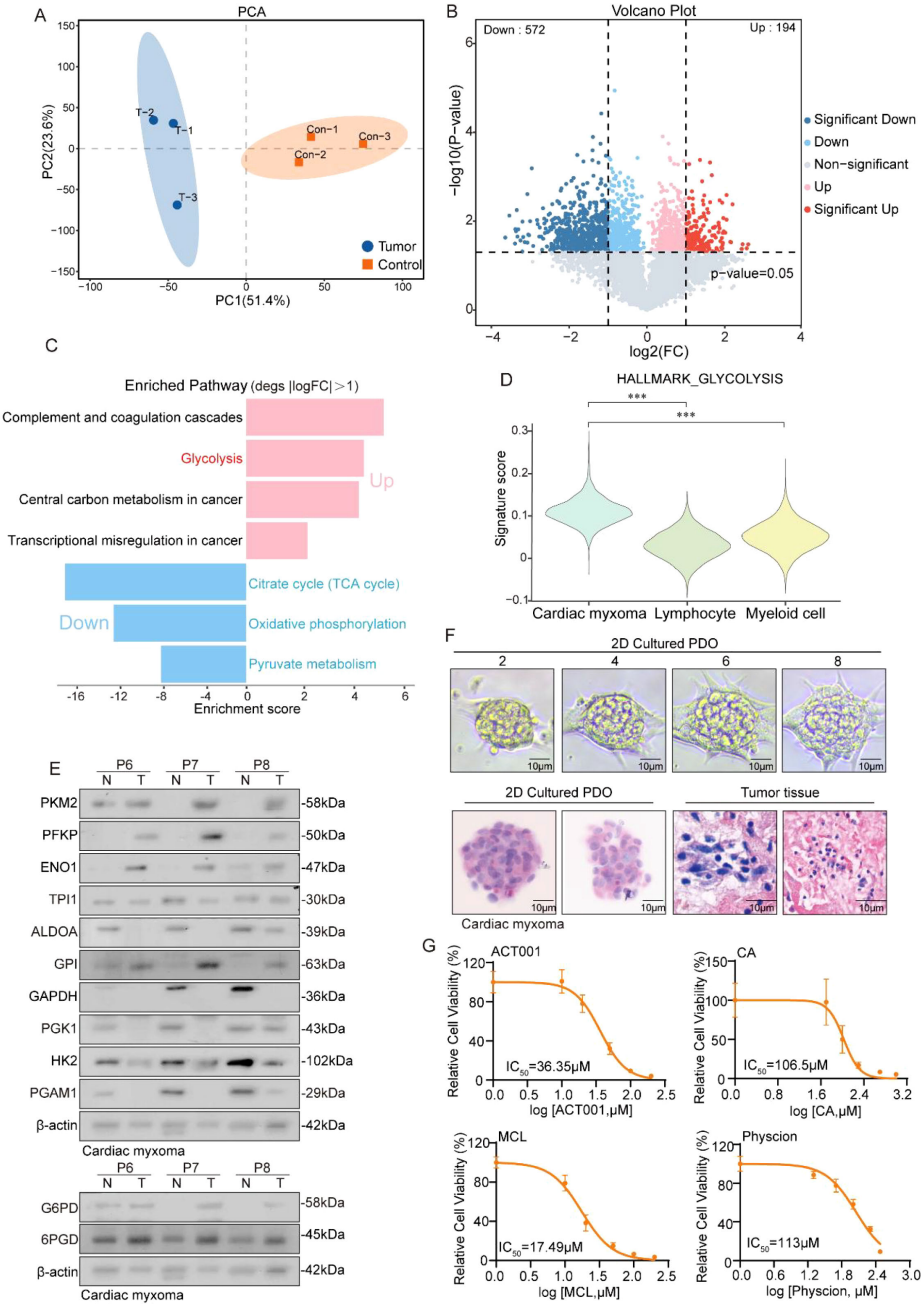
Proteomics reveals a robust glycolytic pathway in CM. **(A)** PCA plot shows the dimensionality reduction of tumor samples and adjacent tissue samples. **(B)** Volcano plot showing the differentially expressed genes between CM tissue and adjacent tissue. **(C)** The histogram displays the enrichment results of upregulated and downregulated genes, with the horizontal axis representing the enrichment score. **(D)** Violin reveals higher glycolysis scores in tumor cells. **(E)** Western blot experiment shows the protein expression levels of glycolysis and its bypass pentose phosphate pathway metabolic enzymes. **(F)** HE staining shows the similarity between cardiac myxoma tissue and organoids. **(G)** Detection of IC50 values of inhibitors targeting the glycolytic pathway and its bypass, the pentose phosphate pathway, in CM organoids.

While RNA sequencing offers valuable insights into CM characteristics, proteins serve as the primary functional molecules in biological processes. Consequently, we conducted a proteomic profiling of three CM and adjacent normal tissue pairs, with PCA analysis revealing high intragroup similarity and significant intergroup differences, confirming sample stability (**Figure 3A**). A differential protein analysis identified 194 upregulated and 572 downregulated proteins in the tumor tissues (**Figure 3B**), while a functional enrichment analysis linked upregulated proteins to complement/coagulation cascades, glycolysis, cancer metabolism, and transcriptional dysregulation. In addition, downregulated proteins were associated with the TCA cycle, oxidative phosphorylation, and pyruvate metabolism (**Figure 3C**). We focused on glycolysis as a key cancer metabolic pathway and observed elevated pathway activity in tumor cells using “HALLMARK_GLYCOLYSIS” scoring (**Figure 3D**), corroborated by our western blot analysis, which showed upregulated glycolytic and pentose phosphate pathway enzymes (**Figure 3E**). Organoid models validated these findings, with HE staining confirming tissue fidelity (**Figure 3F**). Glycolysis inhibitors (MCL, ACT001, CA) and PPP inhibitor physcion effectively suppressed organoid growth and disrupted morphology (**Figure 3G**). Taken together, the above results indicate that glycolytic pathway activation supports CM survival and proliferation.

### CAFs participate in reshaping the extracellular matrix environment and recruiting immune cells to infiltrate

**Figure 4:**
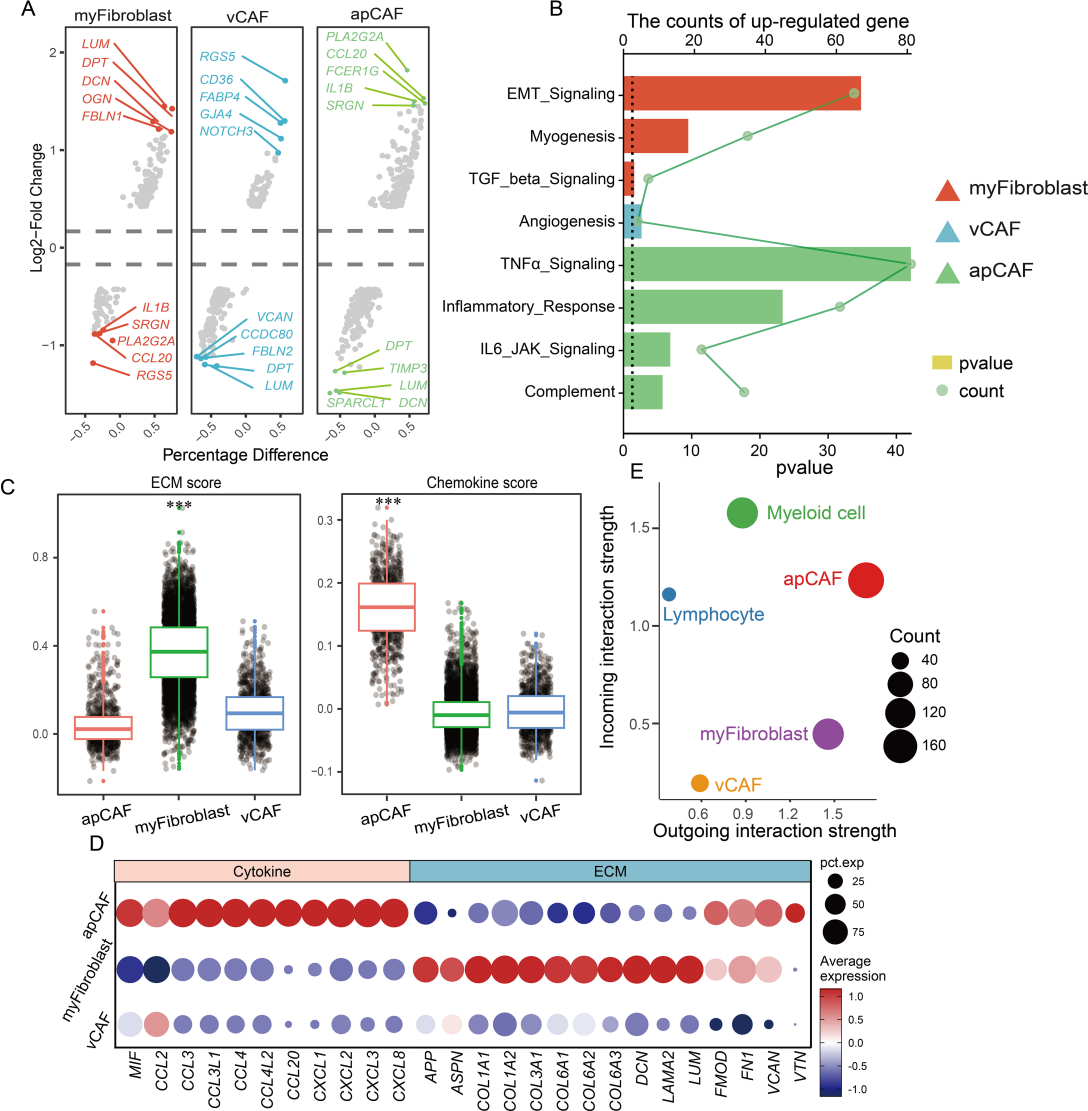
CAF participates in remodeling the extracellular matrix environment and recruiting immune cells. **(A)** Multiple sets of volcano plots demonstrate variations in genetic profiles among different populations of CAFs. **(B)** Bar chart representing KEGG enrichment results, and different colors represent different CAFs. **(C)** Evaluation of CAF population function by scatter plot assessment of ECM and chemokine factor gene set scores. **(D)** Dotplot displaying the expression of ECM and chemokine factor gene sets. **(E)** Cell communication analysis reveals the strength of signal transmission between immune cell populations and CAF populations.

While the distinct spatial distribution of CAF subtypes exhibits specialized features, though their functional consequences in CM microenvironment development remain poorly understood. Therefore, this study pursued a deeper characterization of CAF populations was pursued. Differential gene analysis revealed subtype-specific expression profiles across the CAF subgroups (**Figure 4A**). A pathway enrichment analysis of upregulated genes demonstrated functional specialization: Myofibroblasts showed strong associations with EMT, TGF-β signaling, and myogenesis; vCAFs were enriched for angiogenesis-related pathways; and apCAFs were linked to TNFα signaling, inflammatory responses, IL-6/JAK signaling, and complement activation (**Figure 4B**). These findings not only define CAF functional diversity but also highlight their crucial contributions to the formation and progression of CM microenvironments.

Previous studies have established that cancer-associated fibroblasts (CAFs) principally facilitate tumor microenvironment formation through extracellular matrix (ECM) protein and chemokine secretion. e developed ECM- and chemokine-related gene sets for functional evaluation to precisely characterize CAF populations in CM microenvironments (**Figure 4C**). Our analysis demonstrated that myofibroblasts exhibited significantly higher ECM gene scores than apCAF and vCAF, with the marked upregulation of collagen-forming genes (*COL1A1* and *COL3A1*) critically contributing to tissue rigidity (**Figure 4D**). Conversely, apCAFs displayed substantially elevated chemokine gene scores compared to other subtypes, featuring the prominent expression of *MIF*, *CCL*, and *CXCL* family members. Given that chemokines orchestrate immune cell recruitment, these findings suggest that apCAFs may serve as key mediators of immune cell infiltration in CM microenvironments.

**Figure 5:**
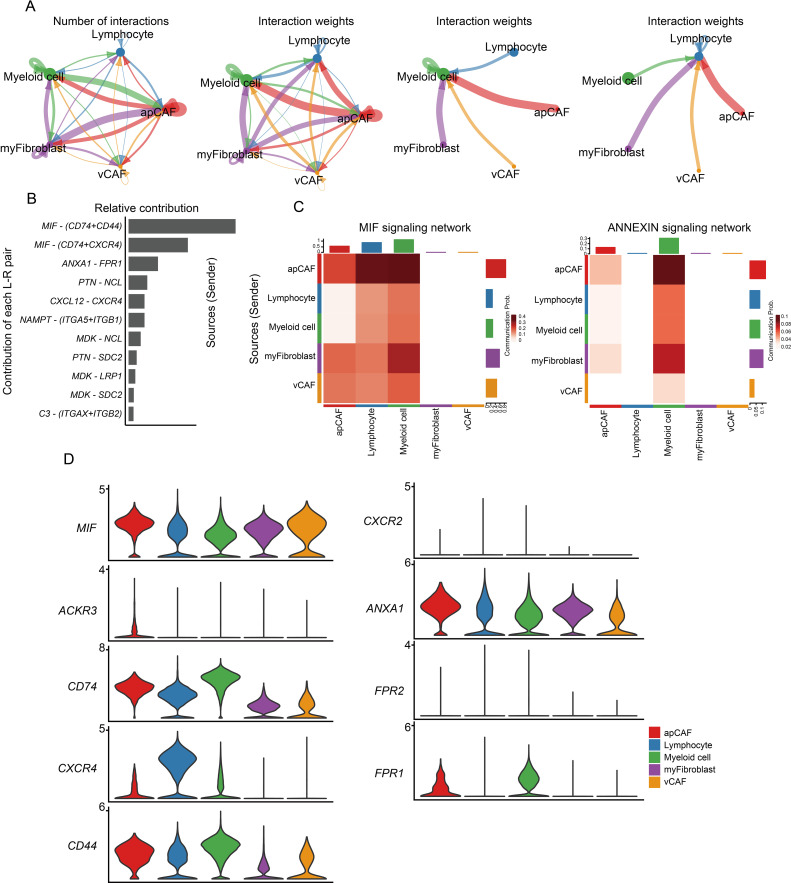
CAF participates in remodeling the extracellular matrix environment and recruiting immune cells. **(A)** The circle diagram illustrates the strength of interaction between immune cells and CAF populations. **(B)** Assessment of ligand-receptor interaction strength. **(C)** Heatmap showing the relative contribution of each cell type based on the computed four-network centrality measures of MIF and ANNEXIN signaling network. **(D)** Expression of MIF and ANXA1 signaling transduction network molecules.

We performed a comprehensive cell communication analysis (17) validate this hypothesis and quantify interaction strengths between CAF subtypes and immune cells (myeloid cells/lymphocytes) in CM microenvironments (**Figure 4E**). The activated protein CAFs exhibited markedly enhanced connectivity with immune cells, particularly myeloid populations. Through systematic evaluation of ligand-receptor pairs, we identified key cytokine networks (*MIF*, *ANXA1*, *PTN*, *CXCL12*, *NAMPT*, *MDK*, and *C3*) driving CM development, with MIF and ANXA1 showing predominant involvement (**Figures 5A, B**). Mechanistically, activated CAFs secrete MIF and ANXA1, which engage CD44 and FPR1 receptors on immune cells (**Figures 5C, D**), orchestrating their recruitment into CM lesions. Collectively, these findings demonstrate that CAFs critically sustain the immune microenvironment through MIF/ANXA1-mediated immune cell recruitment.

### Blocking the signal transmission in antigen-presenting cells leads to the failure of T cell activation

**Figure 6:**
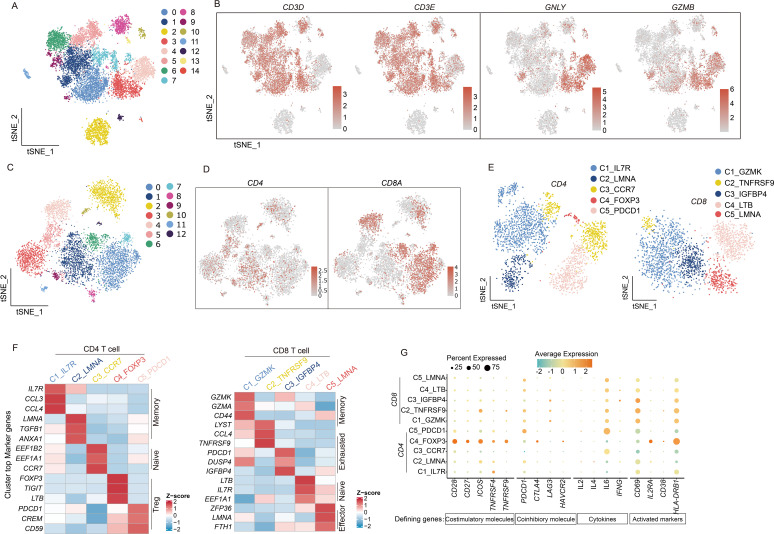
scRNA-seq uncovers the diversity and functional states of T cell subpopulations. **(A)** t-SNE dimensionality reduction graph displaying the distribution of lymphocyte. **(B)** The marker genes *CD3D*, *CD3E*, *GNLY*, and *GZMB* identify T cell and NK cell populations and indicate the proportions of cells. **(C)** The t-SNE graph plot demonstrates main clusters in T cells. **(D)** Featureplot displaying the gene expression of CD4 and CD8A. **(E)** t-SNE dimensionality reduction graph showing 5 subtype in CD4 T cells and CD8 T cells separately. **(F)** Heatmap displaying CD4 and CD8 T cell subtypes and their marker genes. **(G)** Dotplot illustrates the expression of T cell activation factors, activation markers, and immune co-stimulatory molecules. The depth of color represents the level of expression, and the size of the dots represents the percentage of cells expressing the gene.

T cells play critical roles in infection defense, tumor cell elimination (18), and immune regulation. The functional diversity among T-cell subsets enables their comprehensive protection against diverse pathogens and malignant cells. Our analysis classified lymphocytes into 15 distinct clusters (**Figure 6A**), identifying T cells and NK cells using characteristic markers (*CD3D/CD3E* for T cells; *GNLY/GZMB* for NK cells) (**Figure 6B**). Given their predominance, we focused our subsequent analysis on T cell populations (**Figure 6C**), which were further subdivided into CD4+ and CD8+ subsets (**Figures 6D, E**). Marker-based annotation revealed that CD4+ T cells contained Memory (*IL7R*, *CCL3*, *CCL4*; *LMNA*, *TGFB1*, and *ANXA1*), Naive T cells (*EEF1B2*, *EEF1A1*, and *CCR7*), and Treg cells (FOXP3, TIGIT, and *LTB*; *PDCD1*, *CREM*, and *CD59*) (**Figure 6F**, left), whereas CD8+ T cells comprised Memory T cells (*GZMK*, *GZMA*, and *CD44*), Exhausted T cells (*LYST*, *CCL4*, *TNFRSF9*; *PDCD1*, *DUSP4*, and *IGFBP4*), Naive T cells (*LTB*, *IL7R*, and *EEF1A1*), and Effector T cells (*ZFP36*, *LMNA*, and *FTH1*) (19) (**Figure 6F**, right). This functional heterogeneity not only reflects specialized T-cell activities but also implies significant impacts on overall immune system dynamics.

T-cell cytotoxic activity requires prior activation through a dual-signal mechanism. The first activation signal originates from T-cell receptor (TCR) engagement with MHC/antigen peptide complexes, delivering antigen-specific recognition. Costimulatory molecules on antigen-presenting cells concurrently provide the essential second signal. This coordinated dual-signal reception triggers characteristic T-cell activation markers (20).

We examined cytokine secretion profiles (*IL-2*, *IL-4*, *IL-6*, and *IFNG*) (21–24) from activated T cells (*CD69*, *IL-2RA*, *CD38*, and *HLA-DRB1*) (25) to evaluate T-cell activation in the CM tumor microenvironment, revealing consistently low expression levels. A parallel assessment of costimulatory molecules showed similarly reduced expression across both activating (*CD28*, *CD27*, *ICOS*, *TNFRSF4*, and *TNFRSF9*) and inhibitory types (*PDCD1*, *CTLA4*, *LAG3*, and *HAVCR2*) (26) second signal components (**Figure 6G**). These findings strongly indicate a predominantly quiescent T-cell state in CM microenvironments.

### The immunosuppressive landscape of myeloid cells and the SIRPA-CD47 immune checkpoint facilitate immune escape in CM

**Figure 7:**
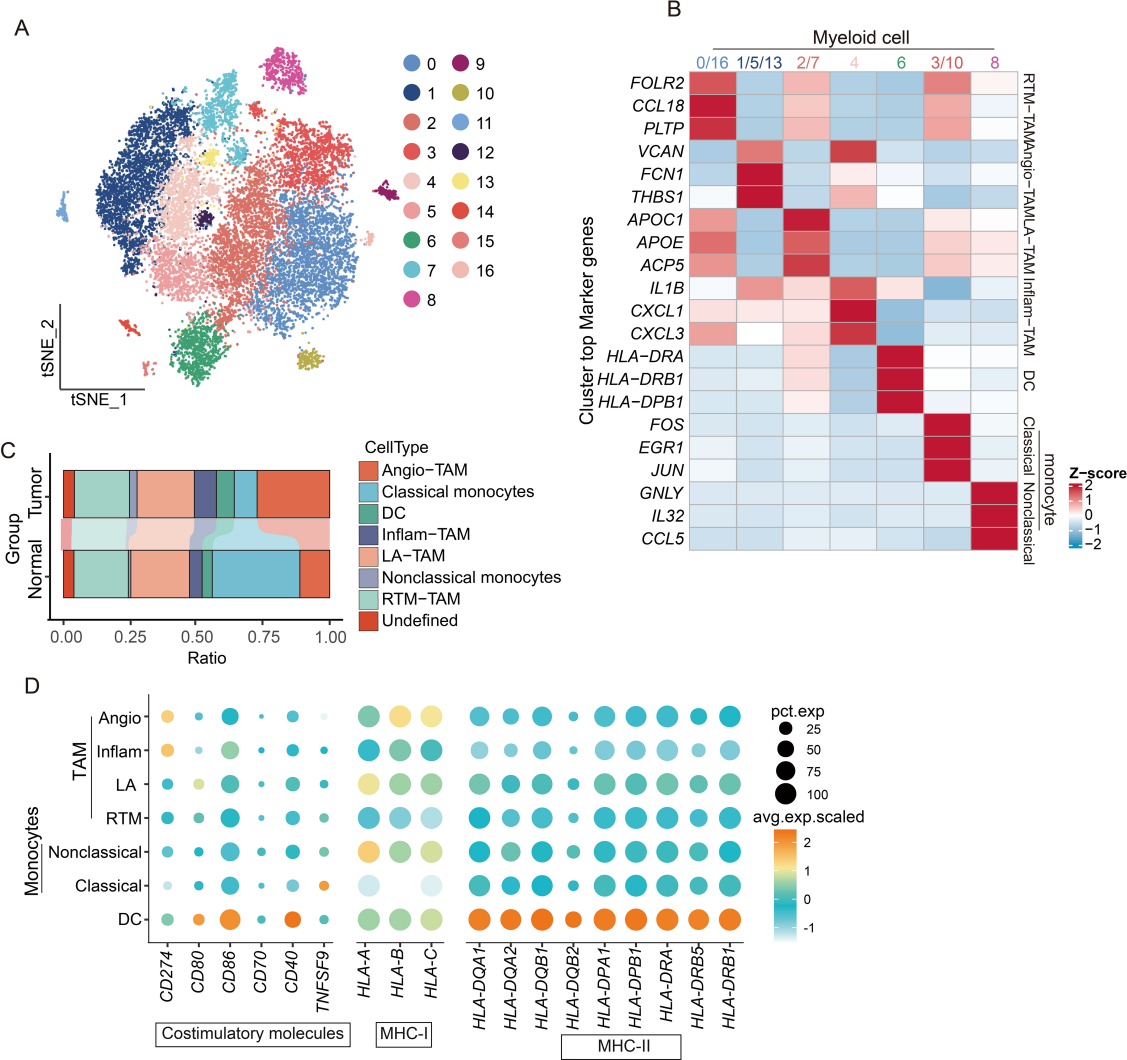
scRNA-seq reveals the diversity of myeloid cell subpopulations and the mechanism of immune activation failure. **(A)** t-SNE dimensionality reduction plot showing clustering and grouping of myeloid cell populations. **(B)** Functional characteristics and marker gene heatmap display of subpopulations of myeloid cells. **(C)** Histogram shows the proportions of different cell types. **(D)** Dotplot graph displays the expression of immune co-stimulatory and MHC molecules in subpopulations of myeloid cells.

We performed a comprehensive clustering analysis of these cells to elucidate the mechanisms impairing myeloid cell-mediated T cell activation, identifying 17 functionally distinct subsets (**Figure 7A**). Tumor-associated macrophages (TAMs) emerged as the predominant population (**Figures 7B, C**), with specialized subtypes displaying unique functional profiles: Resident tissue macrophages (RTM-TAMs) showed elevated CCL18 expression linked to immunosuppression and tumor progression; Pro-angiogenic TAMs (Angio-TAMs) demonstrated marked expression of vascular factors (*VCAN, FCN1, THBS1*); Lipid-associated TAMs (LA-TAMs) exhibited lipid metabolism gene signatures (*APOC1*, *APOE*, *ACP5*); and Inflammatory TAMs (Inflam-TAM) secreted key cytokines (*IL1B*, *CXCL1*, *CXCL3*) that orchestrate immune cell recruitment in tumor-associated inflammation. Notably, antigen-presenting cells showed consistently diminished MHC and costimulatory molecule expression, corroborating our prior observations (**Figure 7D**).

**Figure 8:**
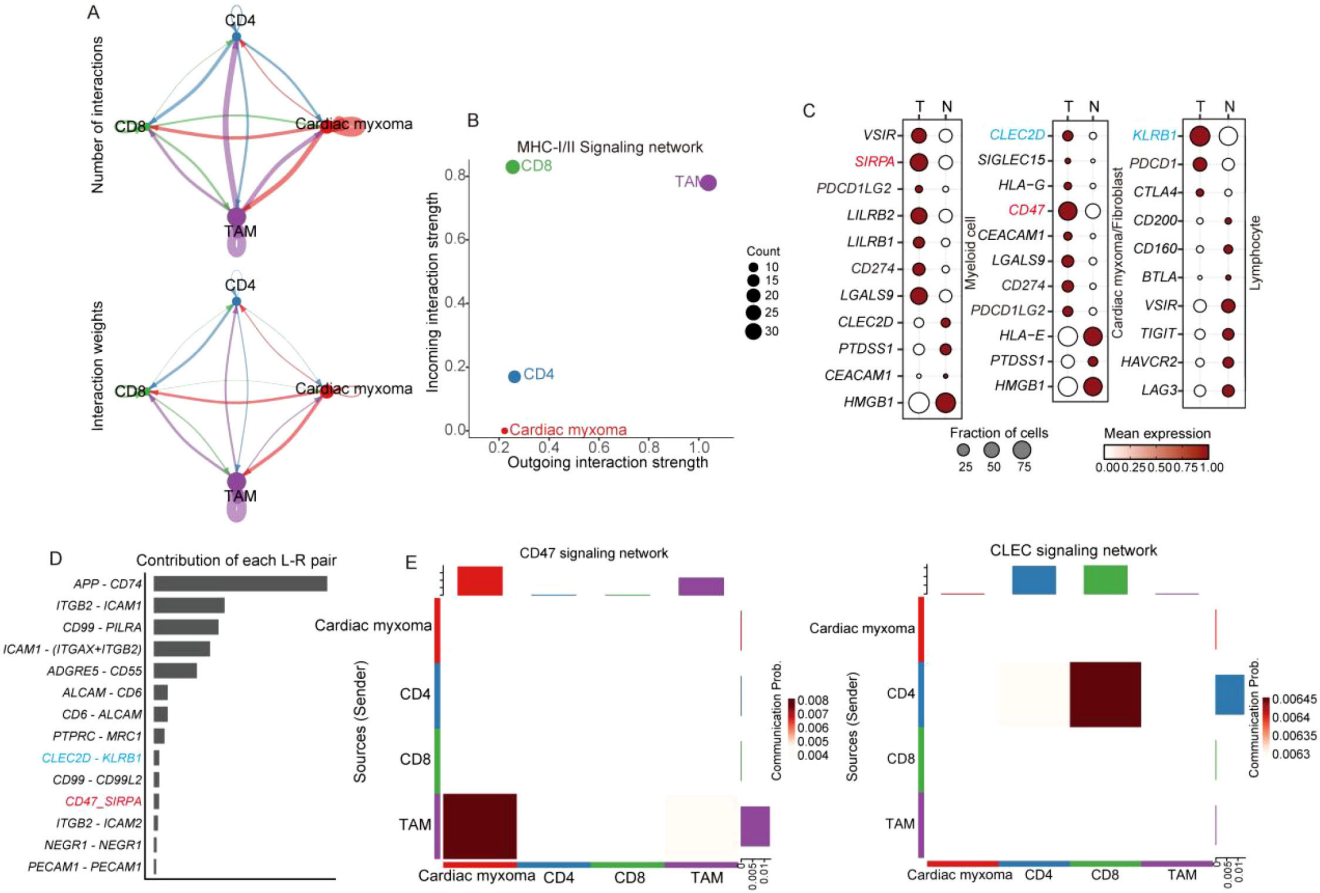
scRNA-seq reveals the diversity of myeloid cell subpopulations and the mechanism of immune activation failure. **(A)** Cell communication analysis methods reveal the strength of interactions among T cell, TAM, and cardiac mucinous tumor cells. **(B)** MHC-I and II signal output, signal reception strength evaluation, with the horizontal and vertical axes representing the strength level, and the size of the dots indicating the number of cells involved in signal transduction. **(C)** Dotplot displaying of the expression of immune checkpoints in different organizations. **(D)** Histograms illustrating the involvement of specific ligand pairs. **(E)** Heatmap illustrating immune checkpoint signaling emission and reception.

Macrophages serve as pivotal antigen-presenting cells in immune responses, especially during tumor–T-cell interactions (27). We systematically analyzed these cellular interactions through our comprehensive communication profiling of four distinct cell populations (**Figure 8A**). Our data revealed that merely 30% of tumor-associated macrophages (TAMs) participated in MHC-I/II-mediated signaling (**Figure 8B**), suggesting an insufficient antigen presentation capacity for effective T cell activation. Further ligand-receptor analysis identified critical immune checkpoint pairs (*CLEC2D-KLRB1* and *SIRPA-CD47*) contributing to cardiac mucinous tumor immune evasion (**Figure 8C**). CLEC2D-KLRB1 interactions mechanistically suppress T-cell effector functions, while SIRPA-CD47 engagement blocks macrophage phagocytic activity, collectively impairing immune signal transduction (**Figures 8D, E**).

### Macrophages tend to polarize toward M2

**Figure 9:**
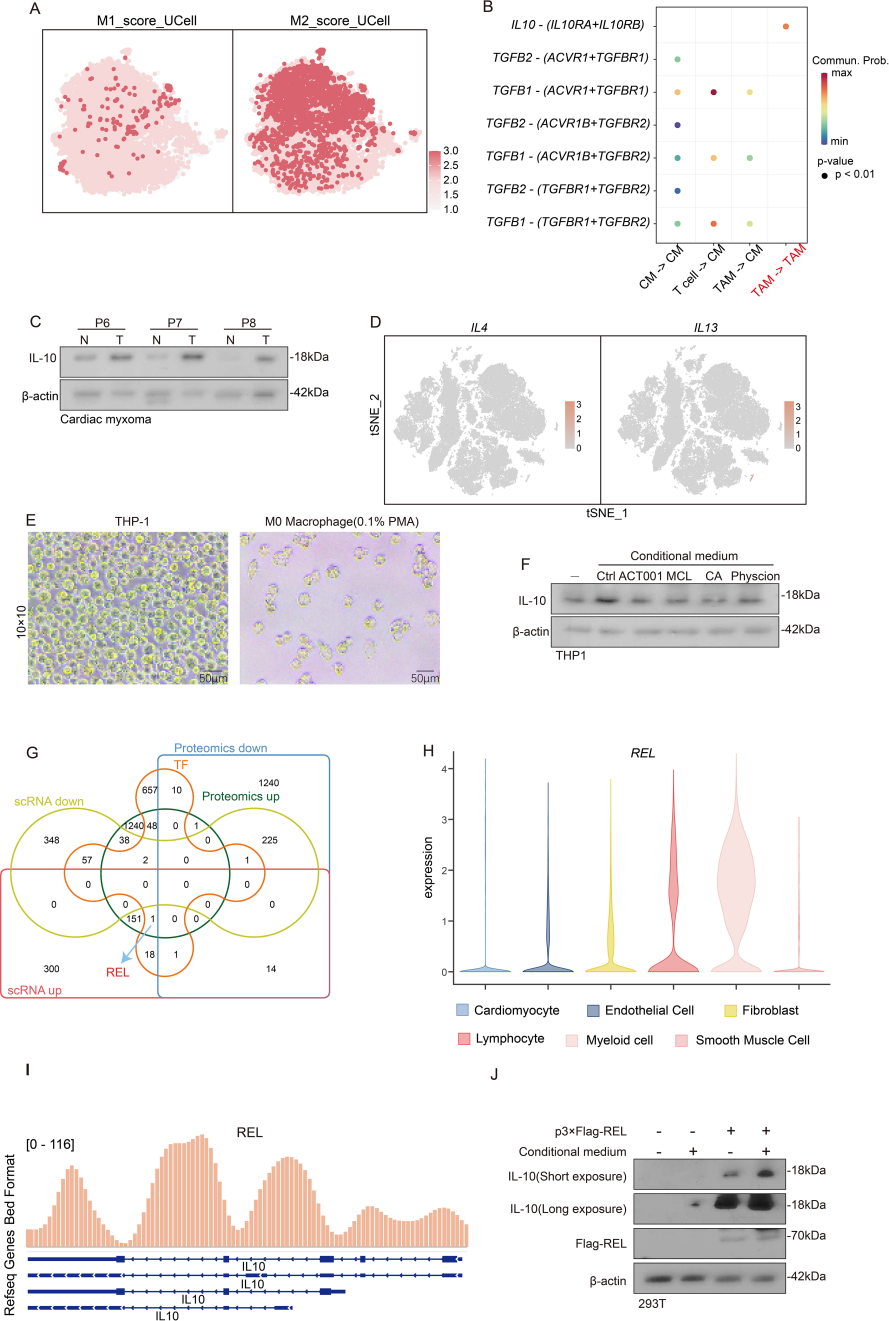
The response of REL to the products of glycolysis metabolism promotes the induction of TAM toward M2 polarization. **(A)** t-SNE dimensionality reduction plot reveals the degree of TAM M1 or M2 polarization via the AUCell scoring. **(B)** Cell communication analysis reveals the degree of interaction between T cells, cardiac myxoma, and TAM. **(C)** Western blot experiment reveals the expression of IL-10 in tumor tissue and adjacent tissue. **(D)** Featureplot displays the gene set of IL-4 and IL-13. **(E)** Observation of THP1 differentiation into M0 macrophages in morphology. **(F)** Evaluate the *IL-10* levels after treating M0 macrophages with conditional culture medium. **(G)** Venn diagram identification of differential genes in scRNA-seq, proteomics and transcription factors. **(H)** Violin plot showing the expression of REL in different subpopulations of myeloid cells. **(I)** ChIP-seq reveals the interaction between *REL* and the *IL-10* promoter. **(J)** Investigate the expression of *IL-10* in M0 macrophages under different conditions of the presence or absence of REL.

Macrophages exhibit plasticity in differentiating into distinct TAM subtypes, which are classically categorized as pro-inflammatory M1 and immunosuppressive M2 phenotypes (28), and are shaped by diverse tumor microenvironmental cues. M1 macrophages demonstrate tumoricidal properties through enhanced immune surveillance and antitumor responses, whereas M2 macrophages facilitate immune tolerance by supporting tissue remodeling and tumor progression (29). We established M1/M2 signature gene sets and implemented AUCell scoring analysis to characterize TAM polarization patterns in CM. A quantitative assessment revealed predominant M2 polarization across TAM populations in CM, with significantly elevated M2 scores compared to their M1 scores (**Figure 9A**). Because M2 macrophages typically mediate immunosuppression via IL-10 and TGFB1 secretion, we employed CellChat analysis to delineate TAM-mediated inhibitory networks (**Figure 9B**). Our western blot validation confirmed substantial IL-10 overexpression in tumors versus adjacent normal tissues (**Figure 9C**). These findings collectively identify the presence of an M2-polarized TAM dominance in CM that orchestrates IL-10-dependent immune suppression.

### Glycolytic metabolites induce the polarization of M2 macrophages

While IL-4 and IL-13 are established inducers of M0-to-M2 macrophage polarization (30), our single-cell analysis revealed remarkably low expression of these cytokines across all CM microenvironmental cell types (**Figure 9D**), implying the existence of alternative M2-polarizing mechanisms in CM. Given the established metabolic regulation of immune function, we noted CM’s distinctive glycolytic hyperactivity (**Figures 3C, D**), which prompted our hypothesis that glycolytic metabolites drive TAM polarization and subsequent IL-10-mediated immune evasion. Our experimental validation involved PMA-induced THP-1-derived M0 macrophages (**Figure 9E**) exposed to 1) an untreated organoid-conditioned medium or 2) a medium pretreated with MCL/ACT001/CA/physcion (3:7 dilution with a fresh medium). IL-10 quantification via Western blot demonstrated that while the control medium robustly elevated IL-10 expression-indicative of M2 polarization, all drug-treated media significantly attenuated this effect (**Figure 9F**). These findings indicate that tumor-derived glycolytic metabolites are critical regulators of macrophage IL-10 production in CM.

### REL up-regulates *IL-10* in response to glycolysis metabolites stimulation

Our prior proteomic studies identified transcriptional regulation as a key driver of CM pathogenesis (**Figure 3C**). To elucidate macrophage responses to glycolytic metabolites, we integrated proteomic and scRNA-seq datasets, identifying the transcription factor REL,with a highly expression in Myeloid cell (**Figures 9G, H**). ChIP-seq analysis uncovered REL’s direct binding to the IL-10 promoter (**Figure 9I**), indicating its potential involvement in metabolite-sensing mechanisms. Functional validation using p-3×flag-REL transfected HEK293T cells demonstrated REL’s capacity to upregulate IL-10 expression, with the organoid-conditioned medium further amplifying this effect (**Figure 9J**). These findings suggest that REL is a critical mediator of glycolytic metabolite-induced macrophage polarization through IL-10 transcriptional activation.

## Discussion

This study leveraged scRNA-seq technology to conduct a comprehensive and in-depth analysis of CM tissue. Our work represents the first systematic characterization of CM’s cellular architecture and uncovers its previously unknown heterogeneity and complexity. Moreover, we precisely delineated the biological properties and functional contributions of specific cell populations within this microenvironment, offering novel perspectives on cardiac homeostasis. We identified tumor cells residing within fibroblast populations, providing compelling evidence for a potential common lineage between CM and fibroblasts. To substantiate this hypothesis, we implemented sophisticated temporal analysis approaches to reclassify tumor cells along a developmental trajectory using mesenchymal stem cell markers. The temporal expression patterns of these markers notably showed remarkable concordance with our hypothesis, strongly suggesting that CM tumor cells originate from mesenchymal stem cells.

Investigations of tumor metabolism through the lens of metabolic reprogramming hold significant value in enhancing the current comprehension of tumor cell proliferation mechanisms (31). Our analysis of CM metabolic features revealed a notable discrepancy: the scRNA-seq data demonstrated TCA-cycle upregulation in tumor cells, while proteomic profiling indicated TCA-cycle downregulation. This divergence primarily stems from the distinct molecular layers these techniques examine—while transcriptional changes may suggest pathway activation, subsequent translational regulation and post-translational modification can alter protein abundance (32). These apparent contradictions reflect complementary metabolic adaptations. Although tumor cells indicate TCA cycle activation at the transcriptional level, the overall tumor tissue (comprising multiple cell types) exhibits suppression at the protein level. Crucially, both methodologies consistently identified enhanced glycolytic activity in tumor cells. To confirm these findings, we developed a CM organoid model that recapitulates *in vivo* growth conditions. Treatment with glycolysis and pentose phosphate pathway inhibitors markedly reduced organoid viability, demonstrating tumor cells’ reliance on glycolytic metabolism for energy production and survival.

Our study further characterized fibroblast subpopulations, identifying three principal subtypes: apCAFs, vCAFs, and myofibroblasts. These cellular components are fundamentally responsible for preserving tissue integrity and mechanical strength. In CM specimens, we detected marked reductions in extracellular matrix production, particularly regarding collagen deposition, which critically compromises tissue firmness. This pathological softening predisposes to tissue fragmentation, raising the concerning possibility of embolic events when dislodged fragments enter cardiac circulation. Such emboli may subsequently cause devastating thromboembolic complications, including cerebrovascular accidents.

Our cell communication analysis revealed that apCAFs secrete MIF and ANXA1 to facilitate immune cell recruitment. Nevertheless, CM tissue demonstrates a partial capacity to evade immune surveillance. As primary mediators of immune cytotoxicity, T cells formed the basis for our exploration of CM’s immune evasion mechanisms. Initial assessments showed diminished levels of T cell activation factors and markers, indicative of a quiescent state. Since T cell activation necessitates dual signaling, we noted that while the first signal initiates the process, secondary signal molecules typically become upregulated—yet our data revealed uniformly low expression of these molecules. This observation suggests impaired first signal transmission as the likely cause of T cell dysfunction. Supporting this hypothesis, cell communication analysis demonstrated weak interactions between T cells and myeloid-lineage antigen-presenting cells (APCs). A further investigation identified as a tumor cell CD47 binding to APC surface SIRPα as a potential mechanism suppressing phagocytic activity, offering initial insights into CM’s immune escape strategy. Additionally, our lymphocyte profiling confirmed the presence of NK and B cells, although their scarcity relative to T cells precluded detailed analyses in this study. Importantly, their limited abundance does not diminish their biological significance—NK and B cells may play distinct roles in immune modulation and disease pathogenesis. Future studies should comprehensively examine these cells’ functional heterogeneity.

Tumor-associated macrophages (TAMs) represent the predominant population among antigen-presenting cells, with their dual polarization states dictating either protumoral or antitumoral effects. Our AUCell scoring analysis revealed a predominant M2 polarization state of TAMs within the CM microenvironment. Conventionally, M2-polarized TAMs facilitate tumor immune evasion through IL-10/TGFB1 secretion or ARG1 overexpression (33). Intriguingly, we identified that TGFB1 primarily targets tumor cells rather than immune cells (34), potentially reflecting tumor cell origins. ARG1 catalyzes arginine conversion to ornithine and urea, whereas ornithine directly suppresses T cell cytotoxicity (34) - contrasting with our earlier observation of TAM-mediated antigen presentation impairment. IL-10 exhibits broader immunosuppressive effects, inhibiting both T cell cytokine production and antigen presentation across macrophages, dendritic cells, and B cells (35), aligning perfectly with our findings. We subsequently investigated macrophage polarization in response to glycolytic metabolites. Proteomic profiling had previously implicated transcriptional dysregulation in CM pathogenesis. Further exploration identified REL as the predominant transcription factor in TAMs. Flag-REL transfection markedly upregulated IL-10 expression, an effect amplified by conditioned medium supplementation, unequivocally establishing the pivotal role of REL in IL-10 regulation.

This study on cardiac myxoma (CM) microenvironments has two key limitations: (1) Its restricted sample size affects its statistical power and generalizability, and (2) it may potentially have selection biases in patient recruitment. Future research should prioritize multicenter collaborations to address these concerns by expanding sample diversity and implementing standardized selection criteria. Advanced methodologies, including propensity score matching and multiomics integration, could enhance the reliability of future results. Validation through independent cohort studies and clinical trials remains essential. Emerging technologies, such as single-cell spatial transcriptomics, may provide deeper insights into CM’s spatial architecture, while CRISPR-based functional assays could verify therapeutic targets. These improvements would strengthen the biological relevance of future findings and support translational applications. While the current work establishes a foundation for understanding CM pathophysiology, subsequent studies must overcome these constraints through rigorous design and innovative approaches to advance diagnostic and therapeutic development for this rare tumor type.

## Conclusions

**Figure 10:**
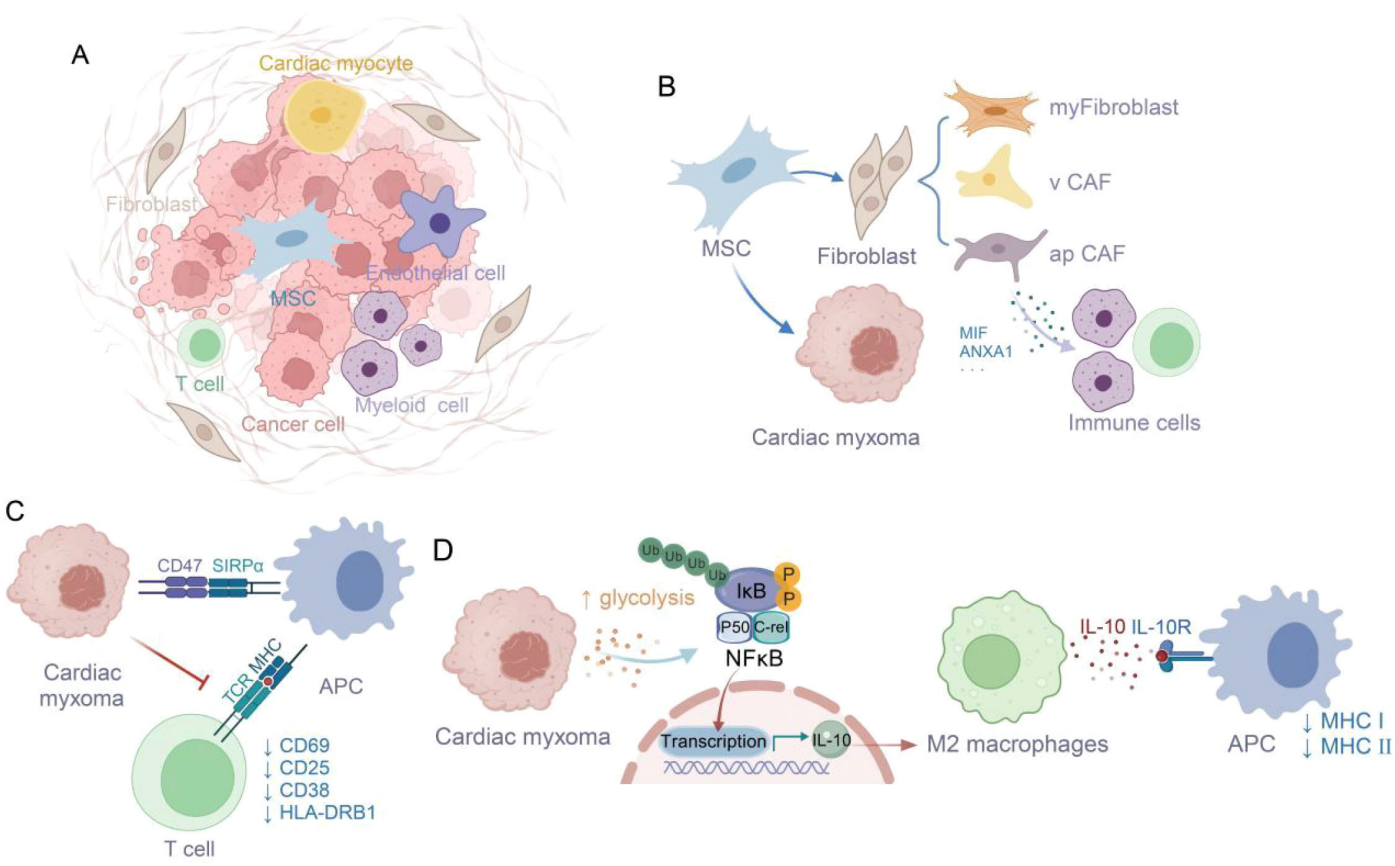
Glycolysis-derived metabolites enhance M2 macrophage polarization and IL-10 secretion, supporting tumor immune escape (36). **(A)** The tumor microenvironment of cardiac myxoma consists of diverse cell types. **(B)** Origin of Cardiac Myxoma Cells from Mesenchymal Stem Cells and Immune Cell Infiltration Induced by apCAF. **(C)** The CD47-SIRPA interaction suppresses macrophage phagocytosis of tumor cells, thereby inhibiting T cell activation. **(D)** Glycolysis affects M2 polarization via NFκB/C-rel signaling pathway.

Collectively, our study has comprehensively characterized the cellular architecture and functional dynamics within the CM microenvironment. We have demonstrated that CM derives from mesenchymal stem cells and sustains its proliferation through glycolytic pathway activation. Moreover, we have uncovered multiple sophisticated strategies employed by tumor cells to circumvent immune surveillance. Notably, the CD47-SIRPα immune checkpoint axis effectively suppresses antigen-presenting cell phagocytosis, while M2-polarized TAMs and their IL-10 secretion collectively impair immune cell functionality. Crucially, we have delineated the intricate crosstalk between tumor metabolism and immune regulation, revealing that the REL transcription factor mediates glycolytic metabolite-induced IL-10 production in M2-TAMs (**Figure 10**) (36). These insights substantially advance our comprehension of CM immune evasion and establish a critical conceptual framework for future therapeutic developments.

## Data Availability

The datasets presented in this study can be available from the corresponding author upon reasonable request. All unique/stable reagents generated in this study are available from the corresponding author with a completed Materials Transfer Agreement.

## References

[1] Rahouma M Arisha MJ Elmously A El-Sayed Ahmed MM Spadaccio C Mehta K. Cardiac tumors prevalence and mortality: A systematic review and meta-analysis. Int J Surg (London England). (2020) 76:178–89. doi: 10.1016/j.ijsu.2020.02.039, PMID: 32169566

[2] Oktaviono YH Saputra PBT Arnindita JN Afgriyuspita LS Kurniawan RB Pasahari D.. Clinical characteristics and surgical outcomes of cardiac myxoma: A meta-analysis of worldwide experience. Eur J Surg Oncol. (2024) 50:107940. doi: 10.1016/j.ejso.2023.107940, PMID: 38219702

[3] Sugimoto T Ogawa K Asada T Mukohara N Nishiwaki M Higami T. Surgical treatment of cardiac myxoma and its complications. Cardiovasc Surg (London England). (1993) 1:395–8. doi: 10.1177/096721099300100418 8076069

[4] Villanueva MT. Bacteria tag tumours for CAR-T cell attack. Nat Rev Drug Discovery. (2023) 22:954–. doi: 10.1038/d41573-023-00181-y, PMID: 37923843

[5] Liu Q Herring CA Sheng Q Ping J Simmons AJ Chen B. Quantitative assessment of cell population diversity in single-cell landscapes. PLoS Biol. (2018) 16:e2006687. doi: 10.1371/journal.pbio.2006687, PMID: 30346945 PMC6211764

[6] Suran M. After the genome-A brief history of proteomics. JAMA. (2022) 328:1168–9. doi: 10.1001/jama.2022.7448, PMID: 36044242

[7] Ruiz-Villalba A Romero JP Hernández SC Vilas-Zornoza A Fortelny N Castro-Labrador L. Single-cell RNA sequencing analysis reveals a crucial role for CTHRC1 (Collagen triple helix repeat containing 1) cardiac fibroblasts after myocardial infarction. Circulation. (2020) 142:1831–47. doi: 10.1161/CIRCULATIONAHA.119.044557, PMID: 32972203 PMC7730974

[8] Yuan P Cheedipudi SM Rouhi L Fan S Simon L Zhao Z. Single-cell RNA sequencing uncovers paracrine functions of the epicardial-derived cells in arrhythmogenic cardiomyopathy. Circulation. (2021) 143:2169–87. doi: 10.1161/CIRCULATIONAHA.120.052928, PMID: 33726497 PMC8169643

[9] Baysoy A Bai Z Satija R Fan R. The technological landscape and applications of single-cell multi-omics. Nat Rev Mol Cell Biol. (2023) 24:695–713. doi: 10.1038/s41580-023-00615-w, PMID: 37280296 PMC10242609

[10] Huang D Ma N Li X Gou Y Duan Y Liu B. Advances in single-cell RNA sequencing and its applications in cancer research. J Hematol Oncol. (2023) 16:98. doi: 10.1186/s13045-023-01494-6, PMID: 37612741 PMC10463514

[11] Liu X Shen H Yu J Luo F Li T Li Q. Resolving the heterogeneous tumour microenvironment in cardiac myxoma through single-cell and spatial transcriptomics. Clin Trans Med. (2024) 14:e1581. doi: 10.1002/ctm2.1581, PMID: 38318640 PMC10844892

[12] Vandereyken K Sifrim A Thienpont B Voet T. Methods and applications for single-cell and spatial multi-omics. Nat Rev Genet. (2023) 24:494–515. doi: 10.1038/s41576-023-00580-2, PMID: 36864178 PMC9979144

[13] Natarajan KN Miao Z Jiang M Huang X Zhou H Xie J. Comparative analysis of sequencing technologies for single-cell transcriptomics. Genome Biol. (2019) 20:70. doi: 10.1186/s13059-019-1676-5, PMID: 30961669 PMC6454680

[14] Liu B Li C Li Z Wang D Ren X Zhang Z. An entropy-based metric for assessing the purity of single cell populations. Nat Commun. (2020) 11:3155. doi: 10.1038/s41467-020-16904-3, PMID: 32572028 PMC7308400

[15] Trapnell C Cacchiarelli D Grimsby J Pokharel P Li S Morse M. The dynamics and regulators of cell fate decisions are revealed by pseudotemporal ordering of single cells. Nat Biotechnol. (2014) 32:381–6. doi: 10.1038/nbt.2859, PMID: 24658644 PMC4122333

[16] Zhang X Lan Y Xu J Quan F Zhao E Deng C. CellMarker: a manually curated resource of cell markers in human and mouse. Nucleic Acids Res. (2019) 47:D721–D8. doi: 10.1093/nar/gky900, PMID: 30289549 PMC6323899

[17] Jin S Guerrero-Juarez CF Zhang L Chang I Ramos R Kuan CH. Inference and analysis of cell-cell communication using CellChat. Nat Commun. (2021) 12:1088. doi: 10.1038/s41467-021-21246-9, PMID: 33597522 PMC7889871

[18] Li C Jiang P Wei S Xu X Wang J. Regulatory T cells in tumor microenvironment: new mechanisms, potential therapeutic strategies and future prospects. Mol Cancer. (2020) 19:116. doi: 10.1186/s12943-020-01234-1, PMID: 32680511 PMC7367382

[19] Rahal OM Wolfe AR Mandal PK Larson R Tin S Jimenez C. Blocking interleukin (IL)4- and IL13-mediated phosphorylation of STAT6 (Tyr641) decreases M2 polarization of macrophages and protects against macrophage-mediated radioresistance of inflammatory breast cancer. Int J Radiat OncologyBiologyPhysics. (2018) 100:1034–43. doi: 10.1016/j.ijrobp.2017.11.043, PMID: 29485045

[20] Xiao S Najafian N Reddy J Albin M Zhu C Jensen E. Differential engagement of Tim-1 during activation can positively or negatively costimulate T cell expansion and effector function. J Exp Med. (2007) 204:1691–702. doi: 10.1084/jem.20062498, PMID: 17606630 PMC2118637

[21] Moynihan KD Kumar MP Sultan H Pappas DC Park T Chin SM. IL-2 targeted to CD8+ T cells promotes robust effector T cell responses and potent antitumor immunity. Cancer Discov. (2024) 14(7):1206–25. doi: 10.1158/2159-8290.c.7309449, PMID: 38563906 PMC11215410

[22] Schüler T Kammertoens T Preiss S Debs P Noben-Trauth N Blankenstein T. Generation of tumor-associated cytotoxic T lymphocytes requires interleukin 4 from CD8(+) T cells. J Exp Med. (2001) 194:1767–75. doi: 10.1084/jem.194.12.1767, PMID: 11748278 PMC2193572

[23] Xu E Pereira MMA Karakasilioti I Theurich S Al-Maarri M Rappl G. Temporal and tissue-specific requirements for T-lymphocyte IL-6 signalling in obesity-associated inflammation and insulin resistance. Nat Commun. (2017) 8:14803. doi: 10.1038/ncomms14803, PMID: 28466852 PMC5418621

[24] Hodny Z Reinis M Hubackova S Vasicova P Bartek J. Interferon gamma/NADPH oxidase defense system in immunity and cancer. Oncoimmunology. (2016) 5:e1080416. doi: 10.1080/2162402X.2015.1080416, PMID: 27057461 PMC4801460

[25] Shipkova M Wieland E. Surface markers of lymphocyte activation and markers of cell proliferation. Clin Chim Acta. (2012) 413:1338–49. doi: 10.1016/j.cca.2011.11.006, PMID: 22120733

[26] Takeuchi M Miyoshi H Semba Y Yamada K Nakashima K Sato K. Co-stimulatory and immune checkpoint molecules are important in the tumor microenvironment of Hodgkin-like adult T-cell leukemia/lymphoma. Haematologica. (2023) 108:3496–501. doi: 10.3324/haematol.2023.283163, PMID: 37439334 PMC10690911

[27] Lu M Huang B Hanash SM Onuchic JN Ben-Jacob E. Modeling putative therapeutic implications of exosome exchange between tumor and immune cells. Proc Natl Acad Sci U S A. (2014) 111:E4165–74. doi: 10.1073/pnas.1416745111, PMID: 25246552 PMC4210007

[28] Epelman S Lavine Kory J Randolph Gwendalyn J. Origin and functions of tissue macrophages. Immunity. (2014) 41:21–35. doi: 10.1016/j.immuni.2014.06.013, PMID: 25035951 PMC4470379

[29] Coburn LA Singh K Asim M Barry DP Allaman MM Al-Greene NT. Loss of solute carrier family 7 member 2 exacerbates inflammation-associated colon tumorigenesis. Oncogene. (2019) 38:1067–79. doi: 10.1038/s41388-018-0492-9, PMID: 30202097 PMC6377304

[30] Scott TE Lewis CV Zhu M Wang C Samuel CS Drummond GR. IL-4 and IL-13 induce equivalent expression of traditional M2 markers and modulation of reactive oxygen species in human macrophages. Sci Rep. (2023) 13:19589. doi: 10.1038/s41598-023-46237-2, PMID: 37949903 PMC10638413

[31] Jiang Y Yan B Lai W Shi Y Xiao D Jia J. Repression of Hox genes by LMP1 in nasopharyngeal carcinoma and modulation of glycolytic pathway genes by HoxC8. Oncogene. (2015) 34:6079–91. doi: 10.1038/onc.2015.53, PMID: 25745994 PMC4564361

[32] Zhou B Yin H Dong C Sun L Feng W Pu Y. Biodegradable and excretable 2D W(1.33) C i-MXene with vacancy ordering for theory-oriented cancer nanotheranostics in near-infrared biowindow. Adv Sci (Weinh). (2021) 8:e2101043. doi: 10.1002/advs.202101043, PMID: 34716674 PMC8693041

[33] Lin W Wu S Chen X Ye Y Weng Y Pan Y. Characterization of hypoxia signature to evaluate the tumor immune microenvironment and predict prognosis in glioma groups. Front Oncol. (2020) 10:796. doi: 10.3389/fonc.2020.00796, PMID: 32500034 PMC7243125

[34] Zhong A Chen T Zhou T Zhang Z Shi M. TPD52L2 is a prognostic biomarker and correlated with immune infiltration in lung adenocarcinoma. Front Pharmacol. (2021) 12:728420. doi: 10.3389/fphar.2021.728420, PMID: 34744715 PMC8567320

[35] Ashenafi S Muvva JR Mily A Snäll J Zewdie M Chanyalew M. Immunosuppressive features of the microenvironment in lymph nodes granulomas from tuberculosis and HIV-co-infected patients. Am J Pathol. (2022) 192:653–70. doi: 10.1016/j.ajpath.2021.12.013, PMID: 35092727 PMC9302207

[36] Shuai J Huiqin L Luowanyue Z Weiping M Ya Z Tianjian C. Generic Diagramming Platform (GDP): a comprehensive database of high-quality biomedical graphics. Nucleic Acids Res. (2024) 53(D1):D1670-D1676. doi: 10.1093/nar/gkae973, PMID: 39470721 PMC11701665

